# Nomogram for Predicting Risk of Esophagogastric Junction (EGJ) Resection During Laparoscopic Resection of Gastrointestinal Stromal Tumors in EGJ: A Retrospective Multicenter Study

**DOI:** 10.3389/fsurg.2021.712984

**Published:** 2021-10-11

**Authors:** Yuting Xu, Lijie Luo, Xingyu Feng, Yensheng Zheng, Tao Chen, Rui Zhou, Yong Li, Guoxin Li, Wei Wang, Wenjun Xiong

**Affiliations:** ^1^The Affiliated Zhongshan Hospital of Guangzhou University of Chinese Medicine, Zhongshan, China; ^2^Guangdong Provincial Hospital of Chinese Medicine, The Second Affiliated Hospital of Guangzhou University of Chinese Medicine, Guangzhou, China; ^3^Guangdong General Hospital, Guangdong Academy of Medical Science, Guangzhou, China; ^4^Department of General Surgery, Nanfang Hospital, Southern Medical University, Guangzhou, China; ^5^The Third Affiliated Hospital of Southern Medical University, Guangzhou, China

**Keywords:** gastrointestinal stromal tumors in esophagogastric junction, nomogram, resection of cardiac structure, logistic regression, laparoscopic surgery

## Abstract

**Background:** The established criteria for determining whether to excise the cardia during laparoscopic surgery for gastrointestinal stromal tumors in the esophagogastric junction (EGJ-GISTs) remain controversial. This retrospective multicenter study was conducted to develop a nomogram for predicting the risk of the cardia excision during laparoscopic surgery for EGJ-GISTs.

**Material and Methods:** We reviewed data from 2,127 gastric-GISTs (g-GISTs) patients without distant metastases in four hospital between June 2012 and June 2020. Of those, according to the including criteria, 184 patients [Guangdong Provincial Hospital of Chinese Medicine (*n* = 81), Nanfang Hospital of Southern Medical University (*n* = 60), Guangdong General Hospital (*n* = 34), and The Third Affiliated Hospital of Southern Medical University (*n* = 9)] with EGJ-GISTs were identified and included in this study. Factors contributing to risk of cardia excision were identified and used to create a nomogram. Nomogram performance was assessed using a bootstrapped concordance index (c-index) and calibration plots.

**Results:** According to the multivariate analysis, the distance from the margin of the tumor to the esophagogastric line (EG-line) (cm) (*OR* = 0.001, *95% CI*: 0.00001~0.056, *P* = 0.001) and tumor size (cm) (*OR* = 14.969, *95% CI*: 1.876~119.410, *P* = 0.011) were significantly related to likelihood of cardia structure excision in laparoscopic surgery for EGJ-GISTs. These two factors were used to generate a nomogram for predicting risk of cardia excision using a logistic regression model; a bootstrapped *C-index* of 0.988 (calibrated *C-index* = 0.987) indicated strong predictive ability, with broad calibration.

**Conclusions:** This nomogram based on distance from tumor margin to EG-line and tumor size may serve as a tool for predicting risk of cardia damage during laparoscopic removal of EGJ-GISTs to aid in selection of surgical methods and preoperative neoadjuvant therapy.

## Introduction

Gastrointestinal stromal tumors (GISTs) are a common form of mesenchymal tumor, with an estimated incidence of ~10–15/1,000,000/year, and the most common site of occurrence being the stomach (50–70%) (1, 2). For localized gastric GISTs (gGISTs), the standard treatment is complete surgical excision of the lesion. Laparoscopic surgery has been widely used due to lower patient trauma and shorter recovery time compared to other approaches. Moreover, the National Comprehensive Cancer Network (NCCN) and the European Society for Medical Oncology (ESMO) have both suggested that laparoscopic resection is feasible for GISTs of suitable location and size (3, 4). However, a laparoscopic approach for gGISTs located in the esophagogastric junction (EGJ-GISTs) remains controversial due to the anatomical complexity of this region and the difficulty of preserving function, such as lower esophageal sphincter pressure, which together make this procedure technically challenging.

An EGJ-GIST is defined as a GIST with an upper border <5 cm from the esophagogastric line (5). In 2018, we introduced four methods of laparoscopic resection: “(1) laparoscopic wedge resection using a linear stapler; (2) laparoscopic complete resection by opening the stomach wall and closing with suture or linear stapler; (3) laparoscopic mucosa-preserving resection; and (4) laparoscopic proximal gastrectomy” (citation). All of these methods have acceptable operative indexes and satisfactory postoperative outcomes (6). Local gastrectomy with cardia preservation and proximal gastrectomy without cardia preservation are the current mainstay treatments. Specifically, wedge resection or resection by opening all layers of the stomach wall are typically recommended treatments, since these methods preserve cardia structure. The cardia structure, which consists of an esophageal sphincter, plays an important role in preventing reflux. Cardia resection is accompanied with reflux esophagitis and other concomitant complications, resulting in serious impacts on the patient's quality of life. Since the advent of imatinib, which can increase survival time for patients with GISTs (7, 8), preservation of cardia function will lead to longer lasting positive effects on quality of life in these patients. Moreover, reduction in tumor size due to imatinib treatment should facilitate easier removal of EGJ-GISTs.

In order to better predict whether cardia could be successfully preserved during laparoscopic resection of EJG-GISTs, we performed a retrospective analysis of EGJ-GIST cases spanning 8 years in our clinic in order to develop a nomogram for use in clinic. Nomograms are graphical prediction models that have been widely used in the prognosis of clinical diseases (9–12). However, no nomogram has yet been developed to predict outcomes of laparoscopic cardia excision in EGJ-GISTs. This study therefore aimed to analyze long-term, retrospective, multi-center data to screen for risk factors associated with cardia excision during laparoscopic resection of EGJ-GISTs using Logistic Regression Analysis. Significant risk factors associated with loss of cardia function due to laparoscopic surgery for EGJ-GISTs could then be used to build a nomogram for prediction of cardia resection based on these risks.

## Materials and Methods

### Patients

We retrospectively collected data from 2,127 gGIST patients without distant metastases who underwent laparoscopic surgery with curative intent in Guangdong Provincial Hospital of Chinese Medicine, Nanfang Hospital of Southern Medical University, Guangdong General Hospital, and The Third Affiliated Hospital of Southern Medical University from June 2012 to June 2020. Of these data, we selected the patients who were diagnosed as EGJ-GISTs. All surgical procedure in the study abided by two oncologic surgical principles: protecting the pseudocapsule integrity and achieving microscopically negative margins on final pathology.

The distance from the margin of the tumor to the EG-line, the tumors locations and growth type were identified based on preoperative endoscopy, abdominal computed tomography scan, magnetic resonance imaging and intraoperative measurement. In the light of the NCCN guideline and our experience, the tumors located in the fundus, greater curvature, or anterior wall of the stomach were classified as favorable site while located in the lesser curvature or posterior wall of the stomach were classified as unfavorable site.

**Figure 1:**
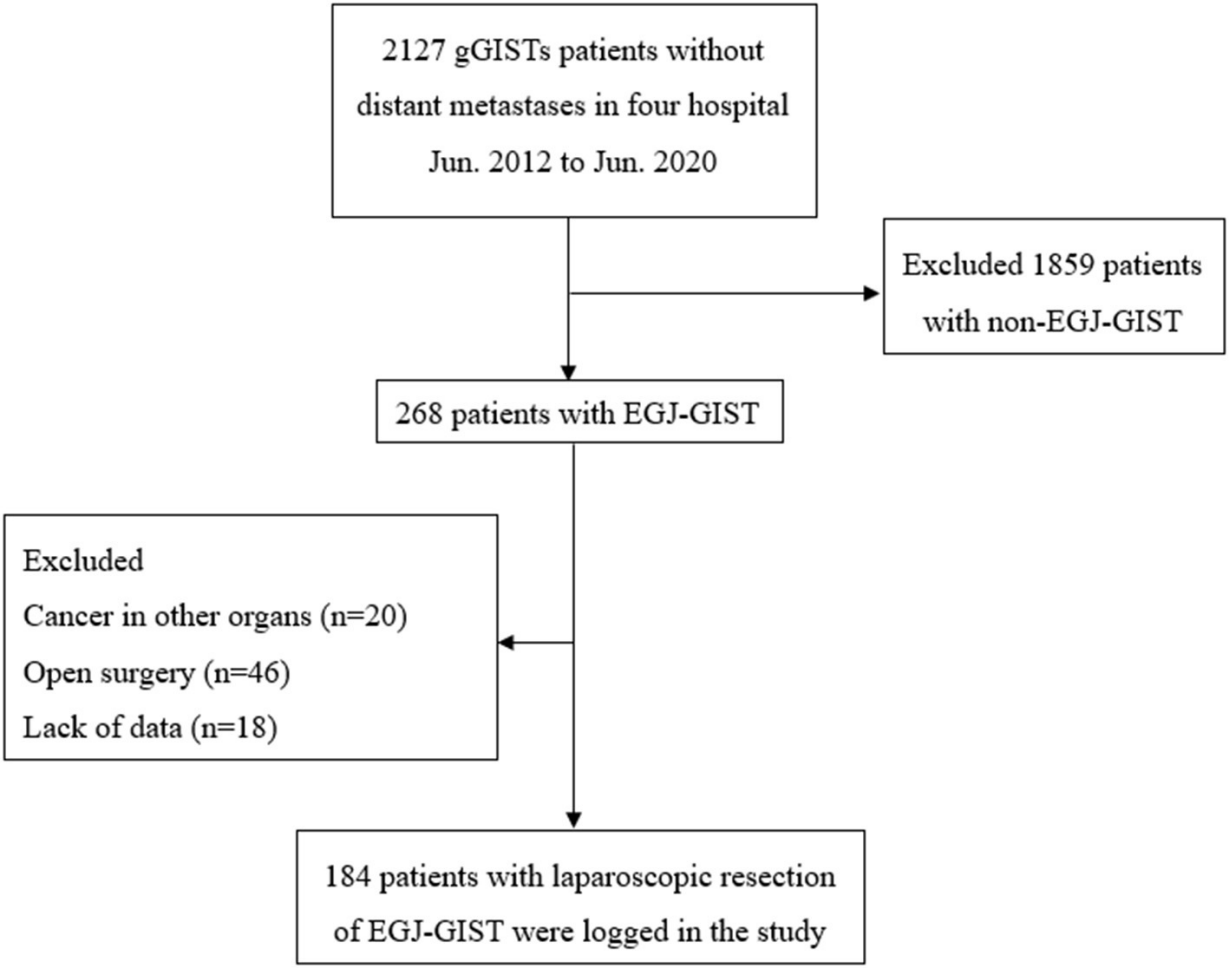
Schematic diagram of patient cohort selection. gGISTs, Gastric gastrointestinal stromal tumors; EGJ-GISTs, Gastrointestinal stromal tumors in esophagogastric junction.

In order to minimize errors, we excluded individuals with cancer in other organs regardless of the severity, lack of data, and those who converted to laparotomy (Figure 1). Approval was obtained from the Institutional Review Board and the registration number was ZE2020-298-01.

### Statistical Analysis

All statistical analyses were carried out using SPSS version 22.0 (SPSS, Chicago, IL, USA) and the R package version 3.6.1 (St. Louis, Missouri, USA). Factors that may affect the success of cardia excision in laparoscopic surgery were evaluated by univariate analyses with a Logistic Regression Model. Risk factors with *P* < 0.05 were included in multivariate analysis. Significant risk factors were used to create a nomogram, which was then applied to predict the risks associated with laparoscopic cardia excision in EGJ-GIST patients. A concordance index (*C-index*) and calibration curve were then used to assess the nomogram. Differences in categorical data between groups were determined by the chi-square test or Fisher's exact test. Log-Rank (Mantel-Cox) was used for comparing the differences of Relapse-Free Survival (RFS) and Overall Survival (OS) between the groups.

## Results

### Patient Characteristics

A total of 184 patients admitted to four hospitals were included in the analyses (Figure 1). The clinical characteristics of these 184 patients who underwent laparoscopic excision of EGJ-GISTs are summarized in Table 1.

**Table 1:** Clinical characteristics of 184 patients of EGJ-GISTs undergoing laparoscopic surgery.

**Factors**	**Number of patients (%)**
**Sex**	
Male	83 (45.1)
Female	101 (54.9)
**Age (years)**, median (IQR)	58.0 (50.0, 66.0)
**BMI (kg/m**^**2**^**)**, median (IQR)	22.6 (20.7, 24.3)
**The distance from the margin of the tumor to the EG-line(cm)**, median (IQR)	3.0 (2.0, 4.0)
**Tumor size(cm)**, median (IQR)	3.5 (2.0, 5.0)
**Growth type**	
Exophytic type	70 (38.0)
Not exophytic type	114 (62.0)
**Tumor location**	
Favorable site	112 (60.9)
Unfavorable site	72 (39.1)

### Risk Factors and Nomogram for Laparoscopic Cardia Excision During Removal of EGJ-GISTs

**Figure 2:**
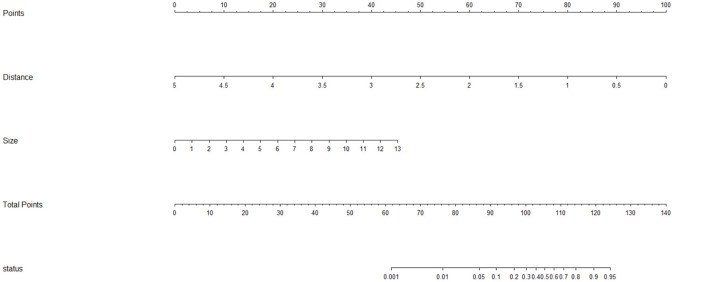
Nomogram for predicting the risk of excising the cardia during laparoscopic surgery for EGJ-GISTs. Distance: Distance from the margin of the tumor to the EG-line (cm); Size: Tumor size (cm); status: probability of EGJ resection.

**Figure 3:**
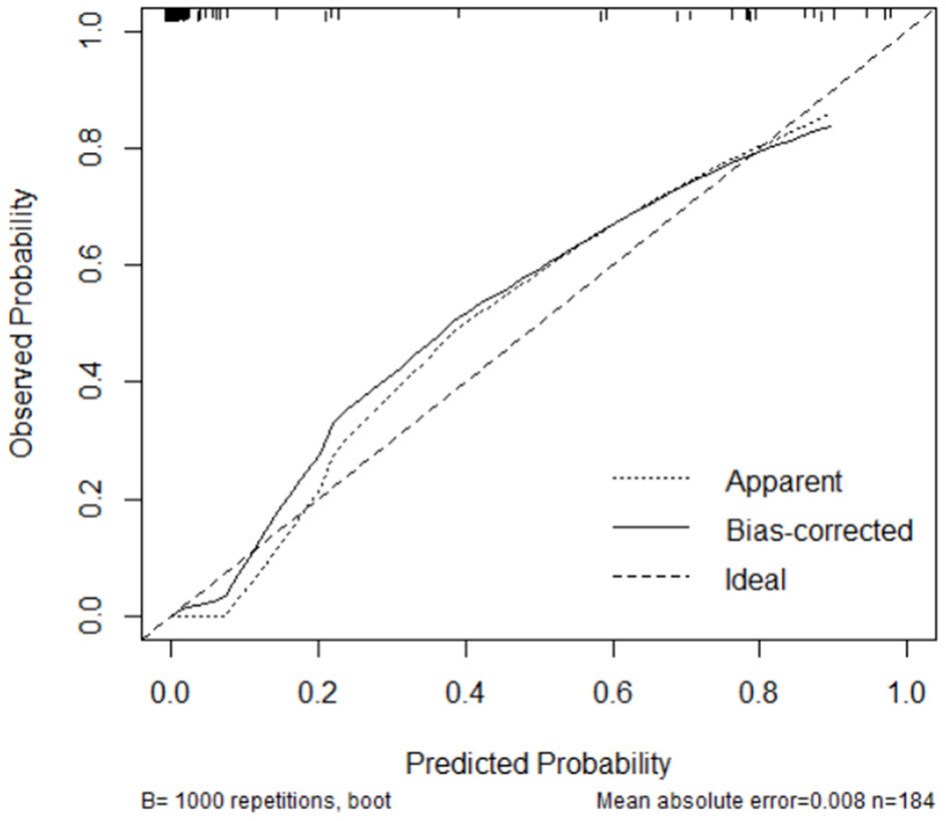
Calibration of the nomogram.

Laparoscopic cardia excision to remove EGJ-GISTs was reported in 18 patients (9.8%). We evaluated the association between clinical factors and cardia excision during laparoscopic tumor resection in the EGJ by univariate and multivariate analyses. Univariate analyses showed that age, distance from the margin of the tumor to the EG-line, tumor size, and tumor location were significantly related to the incidence of cardia excision during laparoscopic surgery for EGJ-GIST (Table 2). Multivariate analysis further showed that distance from the margin of the tumor to the EG-line (*P* = 0.001) and tumor size (*P* = 0.011) were significant factors contributing to the loss of cardia during laparoscopic surgical resection of EGJ-GISTs (Table 2). These factors were subsequently combined to create a nomogram to predict the likelihood of cardia excision during laparoscopic gastric tumor removal from the EGJ (Figure 2). The nomogram had a bootstrapped *C-index* of 0.988 (calibrated *C-index* =0.987) with broad calibration (Figure 3).

**Table 2:** Analyses of the risk of the cardia excision on laparoscopic surgery for EGJ-GISTs.

**Factors**	**Univariate analysis**			**Multivariate analysis**		
	***OR***	***95%CI***	***P***	***OR***	***95%CI***	***P***
Sex (male vs. female)	0.374	0.134~1.044	0.060			
Age (years)	0.396	0.198~0.795	0.009	0.273	0.057~1.295	0.102
BMI (kg/m^2^)	0.720	0.391~1.325	0.291			
The distance from the margin of the tumor to the EG-line (cm)	0.005	0.001~0.047	<0.001	0.001	0.00001~0.056	0.001
Tumor size (cm)	3.316	1.783~6.165	<0.001	14.969	1.876~119.410	0.011
Growth type (Exophytic vs. Not exophytic)	0.598	0.204~1.755	0.349			
Tumor location (Favorable site vs. Unfavorable site)	3.533	1.262~9.896	0.016	2.777	0.292~26.393	0.374

### Postoperative and Oncologic Outcomes

The postoperative complication rate was 9.0% in the patients who the EGJ resection was preserved, while 16.7% in the patients who underwent EGJ resection. There was no significant difference between the two groups (*x*^2^= 0.381, *P* = 0.537). The latest follow-up date for the study was in August 1th 2021. Median follow up was 68.1 months (range 13.7–109.0) in the entire cohort. Neither RFS (*P* = 0.141) nor OS (*P* = 0.673) differed between the patients who underwent EGJ resection and those in whom the EGJ was preserved.

## Discussion

The main purpose of using a nomogram is to provide a visual tool for assessment of whether cardia excision is avoidable if laparoscopic resection is selected for tumors in the EGJ. Surgery is the primary strategy for effective treatment of GISTs, and laparoscopic surgery is widely performed because it is less invasive than laparotomy, thus facilitating quick recovery with minimal cosmetic impacts. However, for GISTs located in the EGJ, laparoscopy is challenging due to several, complex, anatomical factors as well as the difficulty of preserving cardia function. Moreover, these difficulties associated with laparoscopic surgery make this approach controversial, and therefore it is only recommended by guidelines under certain conditions and tumor locations. Recent advances in laparoscopic technique and the emergence of minimally invasive instruments have increased the safety and effectiveness of laparoscopic surgery for EGJ-GISTs (13–15). The key point of laparoscopic surgery of EGJ-GISTs is to achieve microscopic negative margins on final pathology while also retaining the function of the lower esophageal sphincter (LES) without rupturing the tumor pseudocapsule (3, 4). It is therefore beneficial to know the risk of cardia excision in laparoscopic surgery for EGJ-GISTs that accompanies different surgical methods as well as indications for preoperative neoadjuvant chemotherapy.

As a statistical tool, a nomogram prediction model provides a simple and intuitive graph that can accurately estimate the risk of clinical events by integrating the variables that contribute most to these events. Further, nomograms are conducive to individualized treatment, and enable clear and effective communication with patients regarding the risks associated with laparoscopic surgery compared to laparotomy (16–19). In recent years, nomograms have been widely applied to calculate the prognosis of different diseases (9, 10). In this study, we identified the risk factors which may contribute to cardia excision during laparoscopic surgery for EGJ-GISTs through univariate and multivariate logistic regression analyses. Based on our results that the two most significant risk factors were distance from the margin of the tumor to the EG-line and tumor size, we used these measurements to create the first nomographic model, of which we are aware, that forecasts the risk of cardia excision in EGJ-GIST patients.

Han concluded that cardia function could be preserved if the distance between the upper margin of the tumor and the EG-line was at least 1–2 cm and if >50% of the circumference of the EGJ could be reserved after resection (20). In contrast, Kwon proposed that laparoscopic surgery for EGJ-GIST was acceptable if it achieved negative margins (21). In addition, Hiki et al. described Laparoscopic and Endoscopic Cooperative Surgery (LECS) as a treatment for EGJ-GISTs (22), bypassing the difficulty of laparoscopy in order to avoid cardia stenosis and impairment of annular muscle function in the lower esophagus. More recently, the results of a retrospective multicenter study (2017) suggested that laparoscopic and luminal endoscopic cooperative surgery (LECS) was a safe and feasible procedure for the resection of EGJ-GISTs, even in cases when the tumor is located on the EGJ. However, it may be inadvisable if the defect caused by resection is more than one third the circumference of the EGJ (23). In light of previous studies that have sought to minimize the risk of cardia excision, we propose that the nomogram developed in this study can serve as a reliable reference based on tumor size and distance from the upper tumor margin to the EG-line for indication of appropriate surgical methods and neoadjuvant chemotherapy.

In assessing surgical options, it remains controversial whether growth type and tumor location are both important considerations for selection of a treatment strategy and for determining the risk of cardia excision (24, 25). Both NCCN and ESMO recommendations indicate that tumor size, location, growth type, and other factors should be considered in the selection of surgical methods for EGJ-GISTs. Huang et al. enrolled 119 patients with GISTs in the proximity of the EGJ for retrospective review, and concluded that gastric tumors located anywhere but the greater curvature side were more often resected by laparotomy due to the approach angle necessary for linear surgical staplers to access this region of the stomach. However, with advances in technology, since 2016 EGJ-GISTs have been commonly resected laparoscopically (26). Gonzalez et al. reported that LECS for posterior EGJ-GISTs resulted in good efficacy (27). Furthermore, Wang et al. reported performing laparoscopic intragastric surgery for nine cases of cardiac endogenous stromal tumors, all of which resulted in no swallowing disorder or acid reflux symptoms upon follow-up, and therefore concluded that laparoscopic intragastric treatment was safe and feasible for endogenous EGJ-GISTs (28). In addition, techniques such as percutaneous endoscopic intragastric surgery (29) and Single-Incision Laparoscopic Intragastric Surgery (30) have also been reported as safe and feasible for treatment of EGJ-GISTs.

There were several limitations in this study. This was a retrospective study and therefore bias could have been introduced during the analysis; prospective studies are needed to verify our conclusions. In addition, the selection of variables may be incomplete, resulting in limitations for the nomogram prediction model. What's more, we failed to obtain good external verification data for external verification, and only used bootstrap method to verify on the original data set. Despite these limitations, we propose that our nomogram can be informative in the selection of surgical methods and preoperative neoadjuvant therapy.

## Conclusion

The distance from the upper margin of the tumor to the EG-line and tumor size are independent risk factors associated with excision of cardia structure during laparoscopic surgery for EGJ-GISTs. Our nomogram, based on a logistic regression model, may serve as an informative reference tool for the selection of surgical methods and preoperative neoadjuvant therapy through prediction of the risk of damage to the cardia during laparoscopic removal of EGJ-GISTs.

## Data Availability Statement

The original contributions presented in the study are included in the article/supplementary material, further inquiries can be directed to the corresponding author/s.

## Ethics Statement

The studies involving human participants were reviewed and approved by Ethics Committee of Guangdong Provincial Hospital of Chinese Medicine. Written informed consent for participation was not required for this study in accordance with the national legislation and the institutional requirements.

## Author Contributions

YX: project development, data analysis & collection, and manuscript writing. LL and XF: project development and data collection. YZ, RZ, YL, GL, and TC: data collection. WW: project development and manuscript editing. WX: project development, data collection, and manuscript editing. All authors contributed to the article and approved the submitted version.

## Funding

The study was supported by Double First-Class and High-level University Discipline Collaborative Innovation Team Project of Guangzhou University of Chinese Medicine(2021xk48), and the Clinical research project of Guangdong Hospital of traditional Chinese medicine (No. YN10101911).

## Conflict of Interest

The authors declare that the research was conducted in the absence of any commercial or financial relationships that could be construed as a potential conflict of interest.

## Publisher's Note

All claims expressed in this article are solely those of the authors and do not necessarily represent those of their affiliated organizations, or those of the publisher, the editors and the reviewers. Any product that may be evaluated in this article, or claim that may be made by its manufacturer, is not guaranteed or endorsed by the publisher.
